# Identification and characterization of interferon signaling-related microRNAs in occult hepatitis B virus infection

**DOI:** 10.1186/s13148-017-0404-9

**Published:** 2017-09-16

**Authors:** Yiying Wang, Peifu Zhu, Jing Qiu, Jie Wang, Huijuan Zhu, Yinwei Zhu, Lige Zhang, Jie Zhu, Xingxiang Liu, Chen Dong

**Affiliations:** 1 Department of Epidemiology and Statistics, School of Public Health, Jiangsu Key Laboratory and Translational Medicine for Geriatric Disease, Medical College of Soochow University, 199 Renai Road, Suzhou, Jiangsu 215123 China; 2 Guizhou Center for Disease Control and Prevention, Guizhou, China; 3 Zhangjiagang First People Hospital, Suzhou, China; 4 China Huai’an Fourth Hospital, Huai’an, China

## Abstract

**Background:**

Occult hepatitis B virus infection (OBI) is an important risk factor of liver cirrhosis and hepatocellular carcinoma. Type 1 interferon (IFN) signaling-related miRNAs were significantly associated with hepatitis B virus (HBV) infection. However, the characteristics of serum IFN signaling-related miRNAs in OBI remain unclear. Therefore, this study aimed to analyze the expression levels of serum IFN signaling-related miRNAs in OBI and to evaluate their potential values for OBI diagnosis.

**Methods:**

Twenty serum samples for training test (10 healthy controls and 10 OBI patients) and 438 validation serum samples from healthy controls, asymptomatic HBsAg carriers (ASC), and chronic hepatitis B (CHB) and OBI patients were collected. Expression levels of 32 IFN signaling-related miRNAs were analyzed in training and validation sets of samples using RT-qPCR.

**Results:**

Among 32 IFN signaling-related miRNAs, decreased miR-122 levels and increased miR-130a levels were detected in training OBI samples. Furthermore, the results from validation test showed that the mean serum miR-122 and miR-130a level was 2.28 ± 0.96 and 3.11 ± 0.93 in OBI subjects, respectively. Compared to the healthy controls, ASC and CHB patients, miR-122 levels were significantly downregulated, while miR-130a levels were significantly upregulated in OBI patients. ROC analysis indicated that miR-122 + miR-130a could differentiate OBI from healthy controls, ASC, and CHB (≥ 0.87 of AUC).

**Conclusions:**

Our study suggested that decreased serum miR-122 level and increased miR-130a level were significantly associated with OBI. Moreover, a combination of miR-122 and miR-130a could be served as a potential marker for OBI diagnosis.

**Electronic supplementary material:**

The online version of this article (10.1186/s13148-017-0404-9) contains supplementary material, which is available to authorized users.

## Background

Worldwide, hepatitis B virus (HBV) infection remains a major public health issue, with more than 350 million people chronically infected. Infection by HBV can be divided into five clinical categories: asymptomatic, acute, chronic, fulminant, and occult HBV infection (OBI). OBI has been defined as the “presence of HBV viral DNA in the liver (with or without detectable HBV DNA in serum) of HBsAg-negative individuals tested with the currently available serum assays” [1]. Although the mechanism of OBI development is not fully understood, it has been considered that OBI is significantly associated with the pathogenesis of cryptogenic liver disease and increases the risks for liver cirrhosis and hepatocellular carcinoma (HCC) [2, 3].

MicroRNAs (miRNAs) are small ~ 22 nucleotide non-coding RNAs that downregulate gene expression by mRNA degradation or translational repression. Cellular miRNAs can participate in most cellular processes, including cell growth, differentiation, apoptosis, immune reaction, and tumorigenesis [4, 5]. Because most miRNAs are highly stable in circulation and their expression patterns seem to be tissue-specific, serum miRNAs are emerged as new biomarker candidates for the diagnosis of both communicable and non-communicable diseases [6–8].

Endogenous interferons (IFNs) are antiviral and immunomodulatory cytokines that trigger the Janus kinase/signal transducer and activator of transcription (JAK/STAT) pathway with subsequent induction of IFN-stimulated genes (ISGs) [9]. Recently, a growing body of evidences has demonstrated that RNA interference through miRNAs is an inherent component of the IFN-antiviral arsenal [10, 11]. In a multicenter retrospective study, Lin et al. reported that IFN signaling-related miRNAs such as miR-29 family, miR-130a, miR-143, and miR-145 could be used as serum markers for the early screening of HBV-related HCC [12]. Additionally, the previous studies have shown that miR-122, miR-125b, miR-372/373, and miR-146 could influence HBV replication in vitro and were significantly associated with HBV-related cirrhosis and HCC [13, 14].

Considering that type I IFN signaling plays a critical role in HBV infections, we conducted the present study to analyze the expression profiles of serum IFN signaling-related miRNAs in OBI patients and to evaluate their potential values for OBI diagnosis.

## Methods

### Study population

This study was approved by ethics committee of Huai’an Fourth Hospital, Huai’an, China, and written informed consent was obtained from each participant (Ethics Certification Number: hasy/2016ky002). Totally, 117 healthy controls, 107 asymptomatic HBsAg carriers (ASC), 115 persons with chronic hepatitis B (CHB), and 119 OBI patients were recruited from Huai’an Fourth Hospital and Zhangjiagang First People Hospital, respectively. The diagnosis of ASC, CHB, and OBI was based on the “Guidelines for prevention and treatment of chronic hepatitis B, China (2015)” [15]. Participants who had coinfection with human immunodeficiency virus or hepatitis C virus (HCV), autoimmune hepatitis, drug-induced injury, or alcohol abuse were excluded. In addition, we excluded the participants diagnosed with liver cirrhosis or carcinoma in the present study.

### Serum samples preparation

Eight milliliters of fasting blood sample was collected from each participant during his/her first admission to the hospital. Cellular components were removed by two consecutive centrifugation steps (1500 g for 10 min at 4 °C and 2000 g for 3 min at 4 °C, respectively). The supernatant serum was recovered and then stored at − 80 °C.

### Serum RNA extraction

Total RNA was isolated from 300 μL serum with commercial RNA extraction and purification kit (MACHEREY-NAGEL SA, France) according to the manufacturer’s protocol. In order to warrant the consistency in the experimental procedures, exogenous cel-miR-39 was spiked into each serum sample before RNA extraction and then used as an internal control for normalizing miRNA expression level. The concentration of RNA was measured at OD260/280 by a NanoDrop ND-1000 spectrophotometer (Thermo Scientific, Wilmington, Delaware).

### Reverse transcription, real-time quantitative PCR analysis of miRNAs

Reverse transcription was performed with “TaqMan™ Advanced miRNA cDNA Synthesis kit” (Applied Biosystems). The reaction system contained 1.5 μg of total small RNA, 1.2 μl miRNA-RT primers (1 μM), 10 nmol dNTP Mix, and 0.2 μl MMLV reverse transcriptase. TaqMan probe-based microRNA assays (Applied Biosystems) were used to quantify the serum levels of miRNAs in a Q6 Real-Time System (Applied Biosystems) according to the manufacturer’s protocols. As described above, cel-miR-39 TaqManVR miRNA assay (Applied Biosystems) was selected as endogenous control for miRNA expression analysis. The cycle threshold (CT value) was defined as the number of cycles required for the fluorescent signal to cross the threshold in quantitative PCR. The expression of miRNA relative to cel-miR-39 miRNA was reported as dCT (∆CT), which was calculated by subtracting the Ct of cel-miR-39 from the Ct of target miRNA. The relative quantitative of each sample was expressed as log2^-∆CT^.

### Training phase and validation phase study

Totally, 32 miRNAs (Additional file 1: Table S1) which reported to be associated with IFN signaling and HBV infection were selected in the present study. These selected miRNAs were firstly measured using real-time qRT-PCR on a training set of serum samples consisting of ten OBI patients and ten healthy controls. Those up- or downregulated miRNAs in serum (> 2-fold changes) were further validated in another set of serum samples from 107 healthy controls, 107 ASC, 115 CHB patients, and 109 OBI patients.

### Statistical analysis

Data are presented as means ± SD for skewed quantitative data and proportions for categorical data as indicated. Comparisons of differences in the categorical data between groups were performed using *χ* ^2^ test or Fisher’s exact test. Student’s *t* test or one-way ANOVA test was used to analyze continuous variables where appropriate. Pearson’s correlation coefficient analysis was used to evaluate serum IFN signaling-related miRNAs associated with the clinical parameters. Receiver operating characteristic (ROC) and the area under the curve (AUC) were used to calculate the diagnostic values of the examined miRNAs. All statistical tests were two-tailed and a probability level of *P* < 0.05 was considered as statistically significant. Data were analyzed using SAS 9.4 software (SAS Institute, Cary, NC, USA).

## Results

### Demographic and clinical characteristics

The demographic and clinical characteristics of the healthy controls, ASC, CHB, and OBI patients were summarized in Table 1. There were no differences in age and gender among four groups. OBI patients were negative to HBsAg, while ASC individuals and CHB patients were positive to HBsAg. In addition, participants in CHB groups had higher AST and ALT activities than those in healthy controls, ASC group, and OBI group. Compared to the participants with CHB, the lower viral DNA load was detected in OBI subjects.

**Table 1:** Demographic characteristics of the healthy controls and OBI, ASC and CHB patients

Variable	HC *N* = 117	OBI *N* = 119	ASC *N* = 107	CHB *N* = 115	^*χ*2^/F	*P* value	^*χ*2^/F	*P* value^a^
Age (year)	45.58 ± 13.08	42.30 ± 13.60	44.19 ± 15.00	44.40 ± 13.10	1.16	0.3246	–	–
Gender (male)	Male:76	Male:84	Male:76	Male:78	1.26	0.7380	0.27	0.602
BMI (kg/m^2^)	24.83 ± 3.04	24.48 ± 2.94	24.64 ± 4.50	24.38 ± 2.90	0.39	0.7629	0.39	0.763
ALT (IU/L)	22.35 ± 13.94	24.68 ± 11.33	28.42 ± 17.97	99.05 ± 69.47	115.24	< .0001	115.87	0.000
AST (IU/L)	29.36 ± 13.81	28.31 ± 12.28	29.08 ± 14.47	115.52 ± 107.60	70.70	< .0001	71.06	0.000
Anti-HBs(positive)	Positive:64	Positive:20	Positive:25	Positive:13	67.46	< .0001	41.31	0.000
WBC (10^9^/L)	5.03 ± 1.69	5.25 ± 1.92	4.97 ± 1.62	4.84 ± 1.78	1.13	0.3347	1.08	0.358
LYM (10^9^/L)	34.11 ± 8.98	33.49 ± 9.28	34.86 ± 8.82	33.22 ± 9.62	0.70	0.5519	0.84	0.472
HBV-DNA (IU/ML)	ND^a^	2.43 ± 1.34	2.49 ± 2.14	4.20 ± 2.28	30.31	< .0001	30.12	0.000
Genotype		Type B(15)	Type B(15)	Type B(27)				
		Type C(83)	Type C(88)	Type C(88)				
		Type B&C(3)	Type B&C(1)					
		ND^a^(18)	ND^a^(3)					

*ND* not detected, *WBC* white blood cell count, *LYM* lymphocyte count, *HC* healthy controls, *OBI* occult hepatitis B virus infection, *ASC* asymptomatic HBsAg carriers, *CHB* chronic hepatitis B

^a^ *P* value was adjusted for age

### Serum IFN signaling-related miRNAs selection in a training set of serum samples

**Fig. 1:**
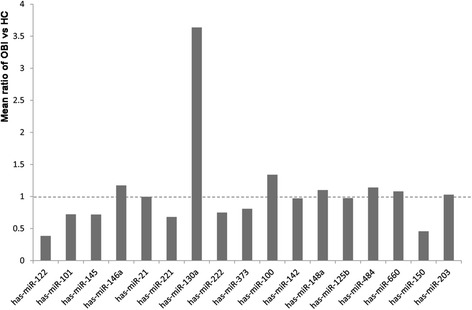
The mean ratio of OBI vs. HC in training samples. Mean ratio value < 1.0: downregulated; mean ratio value > 1.0: upregulated; mean ratio value = 1.0: no changes

We firstly performed the qRT-PCR assay to examine the expression levels of 32 IFN signaling-related miRNAs in the training samples (10 OBI patients and 10 healthy controls). The results showed that serum miR-122 was significantly downregulated, while miR-130a was obviously upregulated in OBI patients when compared with healthy controls (> 2-fold changes. Fig. 1 and Additional file 1: Table S1). No significant differences were observed in the levels of miR-101, miR-145, miR-146a, miR-21, miR-221, miR-222, miR-373, miR-100, miR-142, miR-484, miR-125b, miR-203, miR-660, miR-150, and miR-148a between OBI patients and healthy controls. Additionally, the remained 15 IFN signaling-related miRNAs could not be successfully detected in the serum samples. Therefore, we selected miR-122 and miR-130a as candidate miRNAs for further validation study.

### Serum IFN signaling-related miRNAs in OBI patients validated by qRT-PCR

**Fig. 2:**
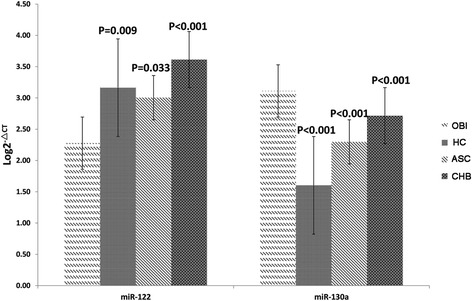
Relative expression levels of serum miR-122 and miR-130a in healthy controls, OBI, ASC, and CHB patients

As the results shown in validated study (Fig 2), the mean serum miR-122 level was 2.28 ± 0.96, 3.16 ± 4.85, 3.00 ± 0.67, and 3.61 ± 0.76 in the groups of OBI, healthy controls, ASC, and CHB, respectively. The levels of serum miR-122 were gradually increased from OBI subjects to healthy control, ASC, and CHB patients (Trends F = 4.00 and *P* = 0.046). In contrast, the mean serum miR-130a level was 3.11 ± 0.93 in OBI subjects, which was significantly higher than those in healthy controls (1.60 ± 0.57) and ASC (2.30 ± 0.63) and CHB (2.72 ± 0.60) patients. The levels of serum miR-130a were gradually decreased from OBI subjects to CHB patients, ASC individuals, and healthy controls (Trends F = 48.23 and *P* < 0.001).

### Relationship of serum miRNAs with demographic and clinical parameters

In the present study, we further analyzed the associations of serum miR122 and miR-130a levels with demographic and clinical parameters. The results showed that serum miR-122 was significantly correlated with HBV DNA level. Additionally, serum miR-130a was significantly correlated with AST and ALT levels. However, there were no significant associations between serum miR-130a or miR-122 and other demographic and clinical parameters (Table 2).

**Table 2:** Associations of serum miR-122 and miR-130a levels with the demographic and clinical parameters

Variables	miR-122		miR-130a	
	*r*	*P* value	*r*	*P* value
Age	−0.16	0.1021	0.09	0.3638
Male gender (%)	0.07	0.1746	0.05	0.2748
BMI	− 0.12	0.2183	0.02	0.8493
AST	0.08	0.1126	0.13	0.0007
ALT	0.07	0.1628	0.16	0.0007
HBV DNA	0.45	< .0001	− 0.03	0.7904

### Diagnostic value of serum IFN signaling-related miRNAs for OBI

**Fig. 3:**
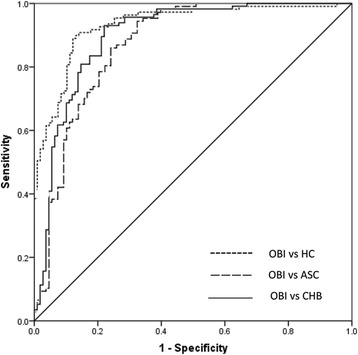
Receiver operating characteristic (ROC) curve analyses using serum miR-122, miR-130a, and miR-122 + miR-130a for discriminating OBI patients from healthy controls and ASC and CHB patients

According to the results presented above, we further tested whether serum miR-122 and miR-130a alone or in combination could differentiate OBI from healthy control, ASC, and CHB patients. The results of ROC analysis showed that as a single marker, miR-122 had the comparable AUC, sensitivity, and specificity to differentiate OBI from other groups (Table 3). Moreover, the ROC curve for miR-122 combination with miR-130a resulted in the highest AUC (0.93 for OBI vs. healthy controls, 0.87 for OBI vs. ASC, and 0.90 for OBI vs. CHB) (Fig. 3 and Table 3). Therefore, it suggested that serum miR-122 combined with miR-130a could be used as a potential marker for OBI diagnosis.

**Table 3:** Comparison of ROC curves for serum miRNA-122, miRNA-130a, and combination of miR-122 and miR-130a

Group	AUC (95% CI)	Best cutoff	Sensitivity	Specificity	*P* value
MiR-122					
OBI vs HC	0.66 (0.58–0.73)	2.39	0.79	0.55	< 0.001
OBI vs ASC	0.75 (0.69–0.82)	2.95	0.59	0.82	< 0.001
OBI vs CHB	0.88 (0.83–0.92)	2.95	0.86	0.83	< 0.001
MiR-130a					
OBI vs HC	0.91 (0.87–0.96)	2.32	0.88	0.89	< 0.001
OBI vs ASC	0.75 (0.69–0.82)	2.95	0.59	0.82	< 0.001
OBI vs CHB	0.67 (0.60–0.74)	3.03	0.60	0.73	< 0.001
MiR-122 + MiR-130a					
OBI vs HC	0.93 (0.90–0.97)	0.43	0.89	0.88	< 0.001
OBI vs ASC	0.87 (0.82–0.92)	0.35	0.94	0.68	< 0.001
OBI vs CHB	0.90 (0.85–0.94)	0.39	0.93	0.78	< 0.001

*HC* healthy controls, *OBI* occult hepatitis B virus infection, *ASC* asymptomatic HBsAg carriers, *CHB* chronic hepatitis B

## Discussion

Although the relationships between serum miRNAs and HBV-related diseases, such as cirrhosis and HCC have been widely investigated previously [12], the information about the characteristics of serum IFN signaling-related miRNAs in OBI patients is limited. In the present study, we observed that serum miR-122 was apparently downregulated, while miR-130a was significantly upregulated in the patients with OBI. Moreover, the combination of serum miR-122 and miR-130a clearly separated the OBI patients from healthy controls and ASC and CHB patients with satisfied specificity and sensitivity.

MiR-122 is a liver-specific microRNA that is involved in a complex signaling network in the biological processes of liver development and differentiation, hepatic lipid metabolism, stress responses, and HCC [16–19]. The results from a Chinese study indicated that silence of miR-122 in HBV transfected Huh-7 cells with antisense miR-122 significantly increased HBsAg and HBeAg secretion [20]. Additionally, Gao et al. observed that suppression of miR-122 induced by HBV infection led to the inactivation of IFN expression, which in turn enhanced HBV replication in Huh7 cells [21]. However, Qiu et al. reported that overexpression of miR-122 could decrease the levels of heme oxygenase-1 (HO-1), while miR-122 inhibitors could increase HO-1 levels. Moreover, overexpression of HO-1 in HepG2 cells could significantly inhibit HBV replication in vitro [22, 23]. In the present study, we observed that serum miR-122 level decreased significantly in the patients with OBI compared to those with ASC and CHB and healthy control. Therefore, in order to unravel the complicated associations between miR-122 and HBV infection, more studies should be performed on this issue in the future.

Our present results showed that serum miR-130a levels were significantly higher in OBI patients than those in healthy controls and ASC and CHB patients. In the previous study, Huang et al. reported that miR-130a attenuated the replication of different genotypes of HBV DNA by reducing HBV transcription and protein synthesis in HBV-replicating hepatocytes [24]. In support of these findings, the results from a Chinese study suggested that overexpression of miR-130a results in increased expression of IFN-α/IFN-β and the IFN-stimulated genes, including MxA, ISG15, and USP18. Moreover, HCV replication was inhibited by miR-130a through restoring type I IFN production [25]. Taken together, we hypothesized that overexpression of miR-130a might be induced by HBV infection and that the increased miR-130a would inhibit HBV replication in the patients with OBI. However, the infection might become chronic if miR-130a could not be successfully stimulated. However, this hypothesis needs to be carefully verified in the future.

## Conclusions

In conclusion, our present study identified that decreased serum miR-122 level and increased miR-130a level were significantly associated with OBI. Moreover, the combination of miR-122 and miR-130a clearly separated OBI subjects from healthy controls, ACS individuals, and CHB patients. More studies focusing on the miRNA-associated pathogenesis of HBV infection are required in the future to further understand the complex relationships between OBI and serum IFN signaling-related miRNAs.

## Additional file

Additional file 1: Table S1. Expression characteristics of selected miRNAs. (XLSX 11 kb)

## Abbreviations

ASC: Asymptomatic HBsAg carriers
CHB: Chronic hepatitis B
HBV: Hepatitis B virus
HCC: Hepatocellular carcinoma
IFN: Type 1 interferon
miRNAs: MicroRNAs
OBI: Occult hepatitis B virus infection
